# Role of NT-proBNP in detection of myocardial damage in childhood leukemia survivors treated with and without anthracyclines

**DOI:** 10.1186/1756-9966-31-86

**Published:** 2012-10-11

**Authors:** Beata Mladosievicova, Dagmar Urbanova, Eva Radvanska, Peter Slavkovsky, Iveta Simkova

**Affiliations:** 1 Institute of Pathological Physiology, School of Medicine, Comenius University, Sasinkova 4, 811 08, Bratislava, Slovak Republic; 2 Department of Neonatology, Faculty Hospital, Nove Zamky, Slovak Republic; 3 Department of Cardiology, Slovak Medical University and National Institute of Cardiovascular Diseases, Bratislava, Slovak Republic

**Keywords:** Cardiotoxicity, Anthracyclines, Natriuretic peptides, Echocardiography, Survivors

## Abstract

**Background:**

Exposure to anthracyclines (ANT) during childhood represents a high risk for development of late cardiotoxicity. Cardiotoxicity is usually detected only when clinical symptoms or progressive cardiac dysfunction have already occurred. Early detection of cardiotoxicity may lead to better therapeutic outcome. N-terminal pro-brain natriuretic peptide (NTproBNP) has been hypothesized to reflect increased left ventricular wall stress before development of echocardiographic abnormalities. The aim of this study was to detect cardiac abnormalities using plasma NTproBNP and echocardiography in asymptomatic childhood leukemia survivors treated with or without cardiotoxic anthracycline therapy.

**Methods:**

Serum levels of NTproBNP were determined in 69 asymptomatic survivors of childhood leukemia treated with or without anthracyclines and in 44 apparently healthy controls. The survivors were divided into two treatment groups: 36 patients after chemotherapy containing anthracyclines (ANT) and 33 patients after chemotherapy without anthracyclines (nonANT). Levels of NTproBNP were measured by using the Elecsys 2010 immunoassay analyzer (Roche Diagnostics). Echocardiography using M-mode, two-dimensional and Doppler measurements were performed on the same day as blood samples were obtained for NTproBNP analysis in survivors.

**Results:**

Serum levels of NTproBNP were significantly higher in the ANT group than in controls (median 51.52 vs 17.37 pg/ml; p=0.0026). Survivors exposed to ANT had significantly increased levels of NTproBNP compared with patients treated without ANT (median 51.52 vs 12.24 pg/ml; p=0.0002). Female exposed and unexposed survivors had significantly higher NTproBNP levels than males. Four of the 36 survivors (11%) treated with ANT and two of the 33 patients (6%) not exposed to ANT had abnormal NTproBNP levels. Although no patient had echocardiographic abnormalities, significant differences were found in values of left ventricular ejection fraction (LVEF) and deceleration time (DT) between survivors treated with or without anthracyclines.

**Conclusions:**

Higher levels of NTproBNP detected in childhood leukemia survivors after low anthracycline cumulative doses might reflect an initial stage of ANT cardiotoxicity before the development of echocardiographic abnormalities. Although the current studies support NTproBNP as one of the best available biochemical markers of late anthracycline cardiotoxicity, a possible strategy toward further improvement and combination with other cardiac biomarkers and novel echocardiographic methods should be explored in additional studies.

## Introduction

Childhood cancer survivors exposed to anthracyclines are at increased risk for premature cardiac morbidity and mortality [1-8]. For 30 years after cancer treatment, survivors are 15 times more likely to experience heart failure than the general population [8]. Cardiac effects of the therapy for acute leukemia in childhood are of particular concern. In more than half of the exposed survivors, cardiotoxic treatment was found to be associated with left ventricular (LV) subclinical structural and functional abnormalities, which can progress to clinically manifested heart failure [9].

Diagnosis of cardiac dysfunction and heart failure after anticancer therapy is based on medical history, physical examination and is further confirmed by other tests, mainly echocardiography. Nevertheless, clinical misdiagnosis is common, particularly in early stages of heart failure. Current monitoring techniques, such as MUGA (Multi Gated Acquisition Scan) or echocardiography, have substantial limitations and detect LV dysfunction only after it had occurred. Cardiotoxicity is usually diagnosed only upon manifestation of clinical signs and symptoms or progressive cardiac dysfunction. Thus new diagnostic tests are required to confirm ventricular dysfunction induced by anticancer therapy .

Novel echocardiographic techniques are promising in evaluating the presence of myocardial structural alterations and subtle myocardial dysfunction induced by anticancer therapy, yet they are not used in routine clinical practice.

Although new cardiac imaging techniques, such as quantitative assessment of ventricular function through measurement of myocardial strain and strain rate can more precisely assess heart structure and function during and early after cardiotoxic therapy, it remains to be proven whether they have the ability to detect early treatment-induced cardiac injury in long-term cancer survivors several years after completion of malignancy therapy. Morevover, the definition of reference range of ventricular strain and strain rate values in normal adults and description of the variability among systems and observers are debatable [10,11].

Early and accurate diagnosis of ventricular dysfunction in asymptomatic cardiac patients may permit a prompt onset of therapy of subclinical cardiotoxicity before the development of life-threatening complications.

This study aims to detect cardiac abnormalities using plasma N-terminal pro brain natriuretic peptide (NTproBNP) and echocardiography in asymptomatic childhood leukemia survivors treated with or without cardiotoxic anthracyclines (ANT).

## Methods

Childhood acute leukemia survivors without any cardiac symptoms were consecutively recruited in the out-patient clinic of the National Cancer Institute, Bratislava, Slovak Republic, from January 2006 to October 2010. A total of 69 survivors of acute leukemia were involved, aged 17–31 years, whose chemotherapy completion dated back for at least 5 years. They had been treated between 1985 and 2005 in a single center – at the Children´s University Hospital, Bratislava. Survivors were divided into two treatment groups: 36 patients who had received chemotherapy containing cardiotoxic anthracyclines (ANT) and 33 patients after chemotherapy without anthracyclines (nonANT) (Table 1). Only one patient was treated with ANT in combination with mediastinal radiation.

**Table 1:** Characteristics of the study participants

	**ANTgroup (N=36)**	**NonANT group (N=33)**	**Control group (N=44)**
***Sex M/F***	19/17	16/17	22/22
***Diagnosis***	ALL (33)	ALL (33)	
***(No. of pts)***	AML (3)		
***Age at diagnosis, yrs***	8 (1–17)	5 (2–10)	
***Median (range)***			
***Time since completion of therapy, yrs Median (range)***	11 (5–22)	15 (6–25)	
***Age at time of the study, yrs***	22 (18–31)	23 (17–31)	23 (20–28)
***Median (range)***			
***Chemotherapy***	Doxorubicin	Cyclophosphamide Vincristine	
	Daunorubicin	L-Asparaginase	
	Epirubicin	Methotrexate	
		6-Mercaptopurine	

Values are presented as median (range).

ALL, acute lymphoblastic leukemia; AML, acute myeloblastic leukemia.

The survivors who had received the cardioprotective agent dexrazoxane were excluded. Furthermore, patients with renal insufficiency, liver dysfunction, abnormal blood pressure, abnormal body mass index and those who were on any current medication, were excluded to avoid possible effects on NTproBNP values.

To establish NTproBNP reference values, we selected a control group of 44 subjects (aged 20–28 years, 50% women) without any known cardiovascular risk factors and no clinical evidence of heart, lung, renal, liver or systemic disease. A blood sample was drawn and stored under the same conditions as in the patients. In this study, our normal values of NTproBNP were different for females (<105 pg/mL) and males (<75 pg/mL) (below 97.5th percentile from controls).

All participants or their guardians gave their written informed consent. The study was approved by the Ethics Committee of the National Cancer Institute and the Faculty of Medicine, Comenius University in Bratislava, Slovak Republic.

All patients were examined by a general cardiologist. The blood samples for immunochemical analysis were obtained at the same day as the echocardiographic measurement was performed.

### Biochemical analysis

EDTA-anticoagulated blood (5 ml) was collected by venous puncture. Fasting was not a prerequisite before sampling. The whole blood was centrifuged for 10 minutes (3500 rpm) within 2 h after sampling. Centrifuged plasma (500 μL) was aliquoted to labeled eppendorf tubes before freezing and stored at −20°C until assayed.

The cardiac biomarker NTproBNP was measured at the Clinical Biochemistry Department, National Cardiovascular Institute, Bratislava, Slovak Republic, within two months after collection. Hemolyzed samples were excluded. Venous blood samples were obtained in the morning and serum concentrations of biomarkers were measured by electrochemiluminescence immunoassay on Elecsys analyzer (Roche Diagnostics). The detection limit for the NTproBNP assay is 5 pg/mL.

We compared the NTproBNP levels between the studied groups exposed and unexposed to ANT and our age- and sex-matched control group.

### Echocardiography

Echocardiography using a GE VIVID 7 machine (GE Ultrasound Europe) was performed in all patients included in the study. Assessment was done by one experienced cardiologist who was unaware of the participants’ treatment status and the NTproBNP value.

Standard techniques were used to obtain M-mode, two-dimensional and Doppler (color, pulse, continuous, tissue) echocardiograms.

Left ventricular (LV) end-diastolic diameter (LVEDD), LV end-systolic diameter (LVESD) and left atrium dimension were measured using standard M-mode methods from parasternal LV long axis images. LV ejection fraction (EF) was calculated by Teichholz formula (M-mode) as well as by the Simpson method from apical view (two-dimensional echocardiography). Systolic LV dysfunction was defined as EF less than or equal to 50%.

Quantification of metric and functional echocardiographic parameters was based on the recommendations of the American Society of Echocardiography´s Guidelines and Standards Committee and the Chamber Quantification Writing Group [12].

Pulsed Doppler traces of the mitral valve inflow were used to extract the ratio of peak early to peak late flow velocities (E/A), E-wave deceleration time (DT), LV isovolumetric relaxation time (IVRT) and were assessed as standard parameters of LV diastolic function. Diastolic LV dysfunction was defined as E/A inversion and DT above 220 ms on the transmitral Doppler curve (impaired relaxation).

The tissue Doppler imaging (TDI) of the mitral annulus from apical four-chamber view provided additional parameters reflecting the global systolic and diastolic function of the LV. Early diastolic velocity (Ea) of the mitral annulus was considered a good indicator of LV myocardial relaxation and diastolic function, and so was the ratio of early diastolic myocardial velocity (Em) and late diastolic myocardial velocity (Am).

Peak systolic velocity at myocardial segments (Sm) was used to assess systolic function. The ratio of early diastolic LV inflow velocity (E) to Ea of the medial mitral annulus (E/Ea) was used for estimation of the LV filling pressure [13].

### Statistical analysis

Continuous variables (echocardiographic parameters) are presented as mean ± SD (standard deviation) and the cardiac biomarker NTproBNP as median and interquartile range. Comparisons between continuous or categorical variables were performed using the Student t-test, Mann–Whitney and Wilcoxon test. Correlations were evaluated with Spearman correlation coefficient. A p-value less than 0.05 was considered statistically significant.

## Results

**Figure 1:**
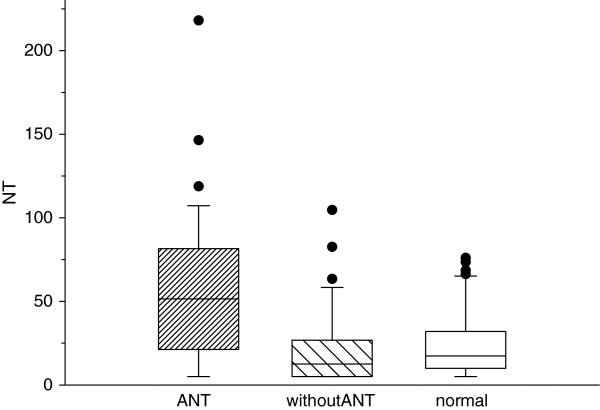
**Comparison of serum levels of NTproBNP in studied groups.** Box plot shows the minimum, maximum, interquartile range (box), and median values for survivors previously treated with and without ANT and for apparently healthy controls. Whiskers above and below boxes indicate the 90^th^ and 10^th^ percentiles. Closed circles outside of boxes indicate outliers.

Serum levels of NTproBNP were significantly higher in survivors treated with anthracylines than in controls (median 51.52 vs 17.37 pg/mL; p=0.0026). Survivors exposed to ANT had significantly increased levels of NTproBNP compared with survivors treated without ANT (median 51.52 vs 12.24 pg/mL; p=0.0002). Levels of NTproBNP in survivors not exposed to ANT compared with controls were not significantly different (median 12.24 vs 17.37 pg/mL; p=0.051) (Figure 1).

Abnormal NTproBNP levels were detected in 4/36 (11%) survivors in the ANT group and in 2/33 (6%) in the nonANT group.

Women exposed to anthracyclines had significantly higher values of NTproBNP than exposed men: median (25th-75th percentiles): 82.6 (51.5-99.1) vs 38.1 (22.2-53.7) pg/mL; p = 0.0031.

Unexposed female survivors had significantly higher values of NTproBNP than unexposed male survivors: median (25th-75th percentiles): 44.6 (21.6-83.2) vs 17.6 (12.5-24.7) pg/mL; p= 0.0039 (Table 2).

**Table 2:** Gender-specific values for NTproBNP (pg/mL) by exposure to anthracyclines

	**Females**	**Males**	**P-value**
**Exposed**	N=17	N=19	
Median (25th-75th)	82.6 (51.5-99.1)	38.1 (22.2-53.7)	0.0031
**Unexposed**	N=17	N=16	
Median (25th-75th)	44.6 (21.6-83.2)	17.6 (12.5-24.7)	0.0039
**Controls**	N=22	N=22	
Median (25th-75th)	28.8 (17.1-44.5)	17.2 (10.3-33.9)	0.12

NTproBNP, N-terminal pro-brain natriuretic peptide. Results are expressed as median and quartiles.

No significant differences in NTproBNP values were found between females and males from control group: median (25th-75th percentiles): 28.8 (17.1-44.5) vs 17.2 (10.3-33.9) pg/mL; p = 0.12.

Although no patient had echocardiographic abnormalities, significant differences were found in values of left ventricular ejection fraction (LVEF) and deceleration time (DT) between survivors exposed and not exposed to anthracyclines (Table 3).

**Table 3:** Echocardiographic parameters in the groups of survivors

	**NonANT group**	**ANTgroup**	**P value**
**LVEF (%) (Simpson)**	69.8 ± 6.4	66.4 ± 4.5	< 0.05
**Sm**	0.12 ± 0.03	0.16 ± 0.16	NS
**E/A**	1.8 ± 0.5	1.7 ± 0.5	NS
**DT (ms)**	195.3 ± 32.9	219.6 ± 55.5	< 0.05
**IVRT (ms)**	72.2 ± 7.9	74.1 ± 7.9	NS
**E/Ea**	6.5 ± 1.4	6.2 ± 1.6	NS
**Em/Am**	2.3 ± 0.7	2.1 ± 0.6	NS
**LVEDD (mm)**	45.7 ± 4.9	46.2 ± 4.2	NS
**LVESD (mm)**	28.1 ± 6.4	29.3 ± 3.5	NS
**LA (mm)**	32.4 ± 3.9	32.5 ± 4.2	NS
**RV (mm)**	26.1 ± 3.2	26.1 ± 3.4	NS

Values are presented as mean ± SD.

NT proBNP values positively correlated with ANT dose (rho = 0.51, p = 0.0028) but failed to correlate with LVEF (rho = 0.1488, p= 0.4245) and DT (rho = 0.1506, p = 0.4269).

## Discussion

Measurement of natriuretic peptides (NP) is routinely used in diagnosis and management of cardiac dysfunction and heart failure [14]. Natriuretic peptides are produced within the heart and released into the circulation in response to increased wall tension, reflecting increased volume or pressure overload. Under pathologic stimuli, the prohormone BNP is synthesized, cleaved to BNP, releasing N-terminal fragment of the brain natriuretic peptide (NTproBNP). Many studies reported that NTproBNP concentrations increased with the severity of ventricular dysfunction and heart failure [13,15-17].

NTproBNP is a promising candidate marker for the exclusion and detection of ventricular dysfunction after potentially cardiotoxic anticancer therapy [2,13,15-28]. Although the role of NTproBNP in the early detection of myocardial damage after anticancer therapy has been evaluated in several studies, the focus was mainly on levels of this biomarker during or only several months after chemotherapy [13,18-20,22,23]. So far only a limited number of studies have investigated the levels of NTproBNP as a marker of late cardiotoxicity occurring several years after completion of chemotherapy in cancer survivors [2,26-28]. In our recent study, we have found significantly elevated NTproBNP levels in childhood leukemia survivors at a median of 10.5 years after completion of anthracycline therapy in comparison with apparently healthy controls [27].

In the present study, this finding was extended to show NTproBNP levels not only in survivors after ANT therapy but also in patients unexposed to anthracyclines. The NTproBNP values in unexposed survivors were found to be comparable to those determined in the control group. According to our information, only one other study reported recently NTproBNP levels in survivors who received ANT compared with patients not receiving these agents [28]. These authors confirmed higher NTproBNP values in the ANT group than in controls yet they found that not only exposed but also unexposed survivors had elevated NTproBNP. They suggest that a chronic inflammatory process may be a predisposing factor of cardiomyopathy in cancer survivors unexposed to anthracyclines. Systemic inflammation in cancer survivors has been of particular concern in recent pathophysiological studies. The discrepancy between the study of Lipshultz et al. [28] and the presented study might be explained by differences in characteristics of the study participants (cancer treatment history, gender, age, body mass index and other risk factors).

In the present study, the detection of cardiotoxicity was performed in childhood leukemia survivors after a low cumulative ANT dose (with median 221 mg/m^2^). So far only few studies have been published that assessed cardiotoxicity after such ANT doses [26,27]. We found significantly higher serum levels of NTproBNP in patients exposed to anthracyclines than in unexposed survivors and controls. These results might reflect anthracycline-induced cardiac abnormalities (such as loss of cardiomyocytes and damage of the remaining cardiomyocytes and other myocardial cells).

The sex-related differences in NTproBNP levels in our patients are consistent with other authors demonstrating that female survivors are more vulnerable to anticancer cardiotoxic and non- cardiotoxic treatments [28].

In the study we found that 11% survivors treated with ANT (with median cumulative dose 221 mg/m^2^) and 6% of patients previously unexposed to anthracyclines had abnormal NTproBNP levels. In the study of Mavinkurve-Groothius et al. [26], abnormal levels of NTproBNP were detected in 13% of 122 asymptomatic survivors of childhood cancer who had received a median cumulative ANT dose comparable to our study. These authors used published reference values for adults derived from a population older or equal to 50 years [29]. The applicability of these cut-off reference NTproBNP values to our adolescent and young adult population may be debatable. In the present study, normal values of NTproBNP were different for females (<105 pg/mL) and males (<75 pg/mL) (below 97.5th percentile from our controls).

Similarly as in some studies, NTproBNP levels were significantly related to cumulative ANT dose in our survivors, yet these concentrations were not correlated with LVEF and DT [23,26].

Given the young age of our survivor population and the rarity of other diseases in young patients, the increased values of NTproBNP found in survivors may provide an useful information on late ANT subclinical cardiotoxicity.

## Conclusions

Higher levels of NTproBNP detected in childhood leukemia survivors after low anthracycline cumulative doses might reflect an initial stage of ANT cardiotoxicity before the development of echocardiographic abnormalities. Although the current studies support NTproBNP as one of the best available biochemical markers of late anthracycline cardiotoxicity, a possible strategy toward further improvement and combination with other cardiac biomarkers and novel echocardiographic methods should be explored in additional studies.

## Abbreviations

A:: Peak flow velocity of late filling; Am:: Late diastolic myocardial velocity; ANT: Group: patients previously treated with anthracyclines; DT:: Deceleration time; E:: Peak flow velocity of early filling; Ea:: Early diastolic annular velocity; EF:: Ejection fraction; Em:: Early diastolic myocardial velocity; IVRT:: Left ventricular isovolumetric relaxation time; LA:: Left atrium dimension; LV:: Left ventricular; LVEDD:: Left ventricular end-diastolic diameter; LVESD:: Left ventricular end-systolic diameter; MUGA:: Multi Gated Acquisition Scan; nonANT:: Patients previously treated without anthracyclines; NTproBNP:: N-terminal pro-brain natriuretic peptide; RV:: Right ventricular dimension; Sm:: Systolic velocity at myocardial segments; TDI:: Tissue Doppler imaging.

## Competing interests

The authors indicated no potential conflicts of interest.

## Authors’ contributions

Conception and design: BM Collection and assembly of data: DU, IS Data analysis and interpretation: ER, PS Manuscript writing: BM, DU Final approval of manuscript: All authors.
